# Dissociated Fear and Spatial Learning in Mice with Deficiency of Ataxin-2

**DOI:** 10.1371/journal.pone.0006235

**Published:** 2009-07-20

**Authors:** Duong P. Huynh, Marwan Maalouf, Alcino J. Silva, Felix E. Schweizer, Stefan M. Pulst

**Affiliations:** 1 Department of Neurology, University of Utah, Salt Lake City, Utah, United States of America; 2 Department of Neurobiology, University of California Los Angeles, Los Angeles, California, United States of America; 3 Brain Institute, University of Utah, Salt Lake City, Utah, United States of America; University of Cambridge, United Kingdom

## Abstract

Mouse models with physiological and behavioral differences attributable to differential plasticity of hippocampal and amygdalar neuronal networks are rare. We previously generated *ataxin-2* (*Atxn2*) knockout mice and demonstrated that these animals lacked obvious anatomical abnormalities of the CNS, but showed marked obesity and reduced fertility. We now report on behavioral changes as a consequence of *Atxn2*-deficiency. *Atxn2*-deficiency was associated with impaired long-term potentiation (LTP) in the amygdala, but normal LTP in the hippocampus. Intact hippocampal plasticity was associated behaviorally with normal Morris Water maze testing. Impaired amygdala plasticity was associated with reduced cued and contextual fear conditioning. Conditioned taste aversion, however, was normal. In addition, knockout mice showed decreased innate fear in several tests and motor hyperactivity in open cage testing. Our results suggest that *Atxn2*-deficiency results in a specific set of behavioral and cellular disturbances that include motor hyperactivity and abnormal fear-related behaviors, but intact hippocampal function. This animal model may be useful for the study of anxiety disorders and should encourage studies of anxiety in patients with spinocerebellar ataxia type 2 (SCA2).

## Introduction

The *ataxin-2* (*ATXN2*) gene belongs to a group of genes, in which expansion of a translated CAG repeat causes neurodegeneration. The function of ataxin-2 is unknown but expansion of the polyglutamine (polyQ) tract from normally 22 to ≥32 repeats causes a late-onset, autosomal dominant ataxia (spinocerebellar ataxia type 2, SCA2), levodopa-responsive Parkinsonism and various cognitive deficits involving mainly executive function and verbal memory [1]–[4].

Ataxin-2 is a cytoplasmic protein that is expressed throughout the brain [5]. Structural analysis and experimental data suggest that ataxin-2 may play an important role in RNA processing. Ataxin-2 contains Like-SM (LSm) domains which are thought to be involved in protein-protein and protein-RNA interactions [6], [7]. Several lines of experimental evidence also implicate a function of ataxin-2 in RNA metabolism. These include observations showing that ataxin-2 is a component of the polysome complex and that it binds to polyA binding protein 1 (PABP-1) in translation initiation [8]. Furthermore, ataxin-2 is a component of stress granules and P-bodies, which are cytoplasmic repositories of untranslated mRNA during cell stress [9], and it interacts with A2BP1/fox-1, a known RNA splicing factor [10], [11].

Although the mouse ortholog of ataxin-2 is more than 90% identical to the human protein, it contains only one glutamine at the site of the human polyQ tract, which suggests that the normal function of ataxin-2 is not dependent on the polyQ tract [12]. Murine ataxin-2 is widely expressed in both neuronal and nonneuronal tissues. However, strong murine ataxin- 2 expression is found in specific neuronal groups such as large pyramidal neurons and Purkinje cells and in subpopulations of neurons in the hippocampus, thalamus, and hypothalamus [5]. In non-neuronal tissues, high levels of ataxin-2 are found in the heart and skeletal muscle. During mouse development, ataxin-2 is expressed as early as embryonic day 8 (E8) in mesenchymal cells and the heart, with a burst of expression at E11 [5]. In humans, high levels of ataxin-2 are found in neurons of the hippocampus and cerebral tissues in addition to Purkinje neurons [13].

To understand the function of ataxin 2, we previously generated *Atxn2* knockout mice using homologous recombination [14]. Despite widespread expression of ataxin-2 throughout development, homozygous *Atxn2* knockout mice were viable, fertile and did not display obvious anatomical or histological abnormalities [14]. A propensity toward hyperphagia and obesity, when fed a moderately-enriched fat diet and subtle motor deficits on the rotarod in late adulthood were observed [14]. These observations were confirmed in an independently generated *Atxn2* knockout model, which in addition demonstrated insulin resistance in *Atxn2*-deficient animals [15].

Several knockout mouse models of other polyQ disease genes have been generated. These include mice deficient for Atxn1, Atxn3 and huntingtin (htt) [16]–[18]. Although htt ko mice were embryonic lethal [17], mouse knockouts of SCA genes survived normally into adulthood. Each line, however, exhibited specific abnormalities such as reduced exploratory behavior and increased levels of ubiquitinated proteins in *Atxn3* ko mice [18], and decreased exploratory behavior, impaired spatial memory in the Morris water maze test, and decreased performance on the rotarod test for *Atxn1* ko mice [16].

The present study describes behavioral and physiological phenotypes of *Atxn2* knockout mice. Our results indicate that *Atxn2* knockout mice exhibit a specific set of behavioral and physiological changes including increased exploratory activity, abnormal fear-related behaviors and impaired long-term potentiation (LTP) in the amygdala.

## Methods

### Ethics Statement

All procedures were approved by the UCLA Institutional Animal Care and Use Committee in accordance with the Public Health Service Policy on Humane Care and Use of Laboratory Animals (PHS Policy) IV.C.5 and USDA Animal Welfare Act Regulations (AWARs) 2.31(d)(5.

### Animals

Generation of an *Atxn2* loss-of-function allele was described previously [14]. Backcrossing mice into a pure C57BL/6J or 129/SvJ background was not possible as litter sizes decreased dramatically once C57BL/6J or 129/SvJ backgrounds exceeded 90%. Therefore, all experiments were carried out in a mixed C57BL/6J:129/SvJ background. Initial breeding pairs (F0) had a defined background of approximately C57BL/6J 50%:129/SvJ 50%. For cohort A, all mice represented F1 animals (B6;129SF1-SCA2^tm1SMP^/J) derived from these breeder pairs. For cohort B, filial matings were continued for 4 generations and F4 mice (B6.129SF4-SCA2^tm1SMP^/J) were used for experiments. In all experiments, littermates were used to represent the other genotypes (wt controls in cohort A, heterozygous knockouts and wt in cohort B).

Mice were housed 4–5 animals per cage under a 12∶12 h light cycle with food and water provided *ad libitum*. The diet was low fat (Purina rodent diet: calories from protein 28.0%, fat 12.1%, carbohydrates 59.8%). For genotyping, about 3–5 mm of tail tip (soft tissue only) was clipped using a sterile, sharp scalpel. DNA was extracted using the Puregene Genomic DNA Purification Kit (Gentra Systems; Minneapolis, MN) and amplified as described previously [14].

To familiarize mice with the investigator (DPH for experiments with cohort A and MM with cohort B), mice were handled for 7 days prior to starting experiments. Each day, the investigator picked up each animal, let it explore the researcher's hand for 2 minutes, and then placed it back into its cage. Handling was repeated 3 times for each animal per day. All mice used in the following experiments were 6 months old and were born within 1–2 weeks of each other.

### Open Field Test

The open field test measures general motor behavior in an arena including measurements for hypo/hyperactivity and environmental habituation. Mice were placed into the periphery of a square clear plexiglass box (41×41×41 cm) equipped with infra-red emitter/detectors linked to a PC and were allowed to explore freely for 20 minutes. Behavior was recorded in X, Y, Z coordinates through infra-red emitter/detectors surrounding the enclosure. The open field was divided into the central zone (28×28 cm) and the peripheral zone. Overall distance and speed were calculated.

Open field tests were performed by 2 different investigators (HPD and MW) using different cohorts of mice. Cohort A consisted of 23 wt and 17 Atxn2 ko mice. Among these were 16 wt female, 7 wt male, 9 Atxn2 ko female, and 8 Atxn2 ko mice. Cohort B consisted of 7 wt, 9 het, and 8 Atxn2 ko mice. There were 3 wt female, 5 het female, 3 Atxn2 ko female, 4 wt male, 4 het male, and 5 Atxn2 ko male mice.

### Morris Water Maze

In the Morris Water Maze paradigm, mice had to escape from a pool of opaque water (kept at 28°C) by locating and climbing onto a plexiglass platform. Swimming trajectories were monitored with a video tracking system (HVS Water Maze Package) that calculated swim speed, trajectory length and average distance from the platform. The latency to find the platform was recorded for each block of the four trials. A total of 8 trials were performed per day. The latencies of the 8 trials were averaged and used for statistical analysis. On the first day, the platform was kept visible to evaluate visual acuity and swimming ability. Mice were given 8 visible platform trials. On the following days, during the hidden platform test, mice learned the location of the escape platform in relation to four external cues in the room. Mice were given 8 hidden platform trials per day from days 2 to 13. Mice were placed into the water facing the wall of the pool and allowed to search for the platform. Trials ended either when a mouse climbed onto the platform or when a maximum of 60 seconds had elapsed. In a block of trials, the starting position was varied among 4 positions. The main measure was the latency to stay in the quadrant with the hidden platform. Probe tests were administered on the 10^th^ and 14^th^ day. During probe tests, the platform was removed from the pool. Mice were started in a position opposite the location of the training platform position and allowed to swim for 60 seconds.

Mice that were floating or showed thigmotaxis during the probe tests were excluded from analysis. Five of 20 wild type mice, but none of the 16 *Atxn2* ko mice exhibited floating and/or thigmotaxis.

### Cued and Contextual Fear Conditioning

Fear conditioning tests were performed by 2 different investigators (HPD and MW) using different cohorts of mice. Cohort A mice consisted of 22 wt and 16 Atxn2 ko. Among these were 13 wt female, 9 Atxn2 ko female, 9 wt male, and 7 Atxn2 ko male. Cohort B consisted of 7 wt, 9 het, and 8 Atxn2 ko mice consisting of 3 wt female, 5 het female, 3 female Atxn2 ko, 4 wt male, 4 het male, and 5 Atxn2 ko mice.

To test aversive memory, wild type and *Atxn2* ko mice were subjected to cued and contextual fear conditioning experiments. In this test, mice were conditioned to associate an auditory cue or environmental contexts with a foot shock. The conditioning chamber was connected to a video monitor that recorded movement in the chamber. During the training phase, a mouse was placed in a conditioning chamber, allowed to explore the testing chamber for 2 minutes before the onset of a discrete conditioned stimulus (CS) in the form of a tone (30 s, 2800 Hz, 80 dB). Within the last 2 seconds of the tone, an unconditional stimulus (US) in the form of a mild foot shock (0.50 mA, 2 s) was delivered. The mouse was allowed to recover for 1 minute, and then a second pair of CS-US was delivered. A total of 3 pairings of the CS-US were delivered during the training phase. After the last CS-US pairing, the mouse was allowed to remain in the conditioning chamber for 1 minute and then was returned to its original cage.

24 hours later, mice were tested for “freezing”. Freezing is defined as a defensive posture, in which no movement except for respiration is observed. For cued conditioning measurements, mice were placed in a novel context and allowed to explore for 2 minutes, followed by exposure to the tone (CS) for 30 s. After a recovery period of 30 s, the tone and recovery period were repeated twice. For measurements of contextual conditioning, mice were placed back into the original conditioning chamber. Freezing was observed for 5 minutes. No shocks were given during testing for cued or contextual freezing. The percentage of total time in which an animal exhibited freezing behavior was recorded.

Response to the shock was measured as described previously [19]. Briefly, full-screen video images for the 2-sec period prior to the shock (baseline) and the 2-sec period during the shock were digitized at 10 Hz using NIH image. X-Y coordinates were collected frame by frame for each mouse by an investigator who did not know the identity of the mouse and the data were imported into Microsoft Excel. Distance traveled measured in pixels between successive frames was computed, and converted into centimeters using known landmark distances in the video frame.

### Startle Test

We used the startle test protocol at the UCLA Behavior core facility to test basic startle mechanisms. Accordingly, mice were placed in a restraint tube mounted on a startle measuring platform and startle response to a loud auditory stimulus was recorded. After a period of 5 min for acclimation, mice were presented with a null trial (no stimulus) followed by 10 trials at startle stimuli with intensities at 75 dB, 80 dB, 85 dB, 90 dB, 95 dB, 100 dB, 105 dB, 110 dB, 115 dB, or 120 dB. At each response, the response expressed relative to the behavior in the absence of auditory stimulation was recorded.

### Hot Plate

The hot plate assay involves placing the animal on a plate at 52.5°C and recording the latency to lick or lift one of the hind paws or to jump with all 4 feet leaving the hotplate to the nearest 0.1 sec. The mice were first habituated to the hot plate before it was turned on by individually placing each mouse on the plate for 30 sec. After habituation, the hot plate was heated to a constant temperature of 52.5°C. Mice were then placed with all four feet onto the hot surface. Once the mouse responded by lifting or licking a hind paw, or by jumping, the mouse was immediately removed from the hot plate and the latency of time to respond was recorded. To prevent burn injury, the maximum time a mouse was allowed to stay on the hotplate was 45 sec [20].

### Light-Dark Box

The apparatus was comprised of a dark and a lighted half. Mice were initially placed in the dark section by blocking the access to the lighter side for acclimation and observed for 5 min. Subsequently, mice were allowed unrestricted access to the lighted compartment for an additional 5 min and the times spent in each compartment were measured.

### Elevated Plus Maze

The apparatus consisted of four elevated arms. Two opposite arms were open and the other two opposite arms were enclosed by walls. The animal was placed in the center of the apparatus and observed for 5 min. Times spent in the central platform, in the open arms and in the closed arms were recorded.

### Conditioned Taste Aversion (CTA)

For CTA experiments, mice were food deprived for 17 days by feeding individual male mice with 2.5 gm of regular and female mice with 1.5 gm of regular chow per day. At the end of food deprivation, the average weight was reduced to about 84.2% of the original weight.

#### Habituation/Training

 Mice were trained for three days to eat food placed in 2 separate food holders. On day 1, mice were trained to eat regular rodent chow in two separate food containers with chow divided equally into 2 food holders. The mouse were allowed to eat for 45 minutes or until the food was gone. On days 2 and 3 of training, mice were trained to eat 20 mg food pellets. Each mouse regardless of gender was fed with 40 chow pellets divided equally into 2 food holders for 45 minutes, and the number of consumed pellets recorded. At this stage, all mice consumed all pellets. Rodent chow, sucrose, and chocolate flavored pellets used in this experiment were purchased from www.ResearchDiets.com.

#### Conditioning (Day 1)

Mice were randomly divided into two groups (group A1 and group A2). Mice from group A1 were fed with 40 sucrose pellets divided equally into 2 food holders. Mice from group A2 were fed with 40 chocolate pellets divided equally into 2 food holders. Each mouse was allowed to consume food for 45 minutes. At the end of this period, each mouse was administered the unconditional stimulus consisting of lithium chloride [20 ul/gm volume, 0.15 M lithium chloride (LiCl) injected IP], and placed back into the cage. One *Atxn2* ko mouse in group A2 was found dead on day 2 after LiCl injection.

#### Testing (Day 2, CS)

Twenty-four hours after conditioning, each mouse from group A1 received 20 chow and 20 sucrose pellets placed in two separate food holders, while mice from group A2 received 20 chow and 20 chocolate pellets. The numbers of food pellets consumed were counted at 15, 30, and 45 min time points for each mouse and the percentages of food pellets consumed at each stage calculated.

#### Neophobia (Day 3)

To test for neophobia to novel foods, the food types were switched between groups. Mice from group A1 received 20 chow and 20 chocolate pellets, while mice from group A2 received 20 chow and 20 sucrose pellets. The number of food pellets consumed at 15 and 45 minutes were counted and percentages of food consumed were calculated.

### Long-term potentiation

To investigate the effect of *Atxn2-*deficiency on long-term potentiation (LTP), hippocampal and amygdala slices from *Atxn2* ko and littermate wild type mice were prepared as described below for LTP measurements. Mice were anesthetized with isoflurane in a desiccator jar inside of a fume hood. After decapitation, the brain was rapidly removed and submerged in ice-cold, oxygenated (95% O_2_ and 5% CO_2_) artificial cerebrospinal fluid (aCSF in mM: NaCl 124, KCl 3, KH2PO4 1.25, MgSO4 2.5, CaCl2 3.4, NaHCO3 26, glucose 10, pH = 7.4). Coronal (amygdala) or horizontal (hippocampus) slices (400 µm thick) were cut on a vibratome and incubated in a submerged holding chamber at room temperature for at least 1 hour before further experimentation. Slices were transferred to a recording chamber on the stage of an upright microscope (Zeiss, Axiovert 200) and perfused with oxygenated artificial CSF at a rate of 1–2 ml/min. Bipolar matrix electrodes (FHC; Bowdoin, ME) were used for stimulation and borosilicate recording electrodes filled with 2 M NaCl (impedance 1–2 MΩ) were used for recording of extracellular field potentials. The stimulation intensity was adjusted to elicit a response with amplitude that was half of the maximal amplitude. Test responses were elicited every 30 seconds and LTP was induced with two high-frequency burst stimulations of afferent fibers (100 Hz for 1 sec, 30 sec interburst intervals). The response amplitude was measured and normalized to the first 5 minutes of each recording.

## Results

### Weight of *Atxn2* ko mice

**Figure 1:**
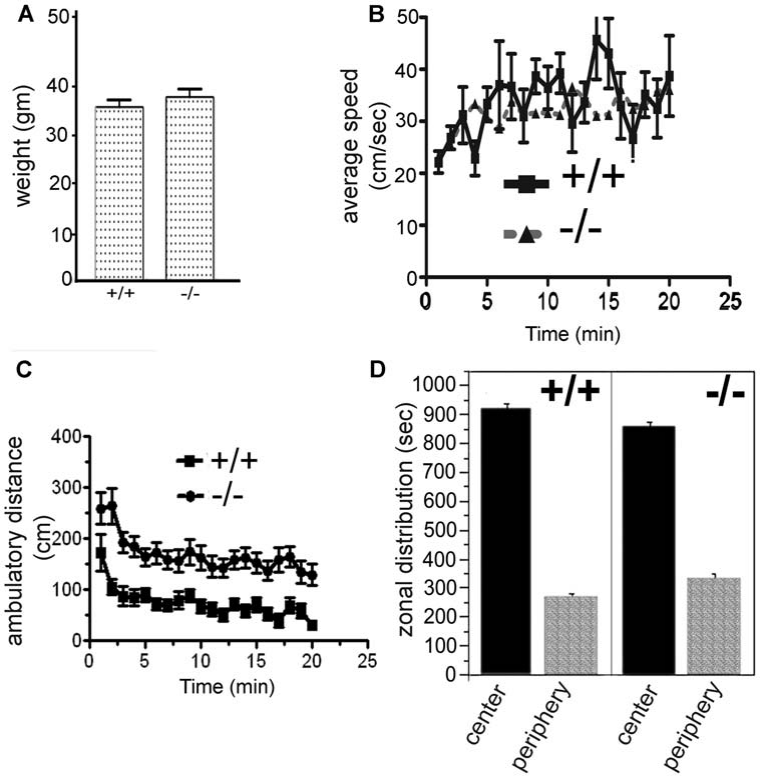
Weight and open cage behavior of mice in cohort A. Atxn2 KO mice exhibited increased motor activity. A) Weight of wild type (+/+, n = 23) and *Atxn2* ko (−/−, n = 16) littermates measured at 6 months of age. Wt: 34.5±1.7 gm (mean±SEM); *Atxn2* ko: 37.8±1.7 gm. B) Average walking rate (cm/sec) of wild type (n = 23) and *Atxn2* ko (n = 16) mice recorded each minute over a 20 minute period. No significant differences between wild type and *Atxn2* ko mice were observed; wt: 33.7±1.4 cm/sec, ko: 31.9±0.8 cm/sec (mean±SEM) (2-way ANOVA, P>0.23). C) Ambulatory distances (cm) traveled by *Atxn2* ko and wild type mice during 20 minutes in the open cage. The average ambulatory distance traveled by *Atxn2* ko mice was significantly greater than that of wild type mice (2-way ANOVA, P<0.0001, Bonferroni's post tests, P<0.0001). D) Environmental habituation of wild type and *Atxn2* ko mice during 20 minutes in the open cage. Both wild type and *Atxn2* ko mice preferred the center zone over the peripheral zone.

Previously, we reported that Atxn2-deficient mice developed obesity when fed a moderately fat-enriched diet (Purina mouse diet 5015: calories from protein 18.2%, fat 25.8%, and carbohydrates 55.9%) [14]. This observation was confirmed by another group for an independently generated *Atxn2* knockout line maintained on a “normal” diet [15]. For experiments described in this report, mice were fed with low-fat Purina rodent chow 5001 (calories from protein 28.0%, fat 12.1%, carbohydrates 59.8%). Using this diet and the housing conditions at the UCLA Behavior Core, weights of *Atxn2* ko mice and wild type mice from cohort A cohort measured at 6 months were not significantly different (Fig. 1A).

### Open cage behavior is abnormal in *Atxn2* ko mice

To measure spontaneous activity and habituation, mice were placed individually into a clear glass cage and motor activity was measured for 20 minutes (Fig. 1B–D). Although both wild type and *Atxn2* ko mice moved at similar speeds when in motion (Fig. 1B), the ambulatory distances traveled by *Atxn2* ko mice were significantly greater than wild type mice (Fig. 1C). *Atxn-2* ko mice traveled an average distance of 163.3±9.1 cm in 20 minutes compared to 73.9±6.5 cm for wild type mice (2-way ANOVA, P<0.0001, Bonferroni posttests, P<0.0001). These differences remain significant when male and female mice were analyzed separately (Fig. S1). Both wild type and ataxin-2 ko mice had similar exploratory patterns as they spent the same amount of time exploring the center zone and very little time in the peripheral zone (Fig. 1D).

A separate but smaller cohort of mice (cohort B) was examined by a different investigator (MM) at 6 months of age. *Atxn2* ko mice moved at a faster speed than wt and heterozygous mice (1-way ANOVA, with repeated measures P<0.0001, Fig. S2). The average speeds were 15.5±1.1, 16.1±1.1, and 21.0±0.8 cm/sec (mean±SEM) for wild type (n = 7), heterozygous (n = 9), and ataxin-2 ko (n = 9) mice, respectively. Similar to cohort A mice, cohort B *Atxn2* ko mice, on average, traveled greater distances (104.7±8.6 cm) than wild type (59.9±6.7 cm) or *Atxn2* heterozygous (58±7.6 cm) mice (one-way ANOVA, P<0.0001). In contrast to cohort A mice, zonal analysis showed that homozygous knockout mice spent significantly more time in the central part of the cage (568.2±50.8 vs. 381.0±57.9 seconds, 2-way ANOVA, P<0.03, Bonferroni posttests, P<0.05) than their wild type littermates (303.0±44.5 vs. 258.7±67.6 seconds). Differences in zonal distributions of cohort A and cohort B may have occurred by chance owing to the smaller number of mice used in cohort B or may reflect subtle differences in testing conditions and mice handling prior to Open Field testing. In all tests, the behavior of heterozygous mice was more similar to wild-type mice than homozygous ko mice.

### Spatial learning is normal in homozygous *Atxn2* ko mice

The Morris water maze (MWM) paradigm is a hippocampus-dependent task. The MWM evaluates the ability of a mouse to locate and remember the position of a submerged platform based on visual clues and requires a normal hippocampus. To investigate spatial learning, we compared performance of wild type and *Atxn2* ko littermates.

**Figure 2:**
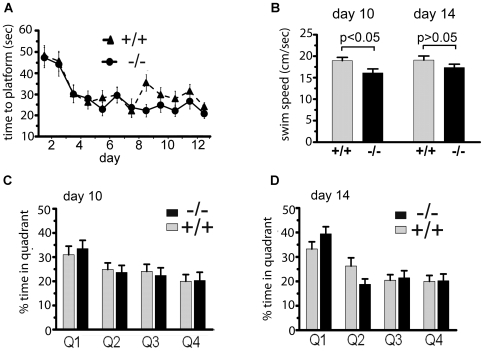
Normal performance of *Atxn2* ko mice in the Morris water maze. A) Escape latency of cohort A mice. Both wild type (n = 16) and *Atxn2* ko mice (n = 16) possessed similar ability to locate the quadrant with the hidden platform. B) Swim speed during probe trials on days 10 and 14. Both genotypes swam equally well during day 14 probe trials, but significant differences were observed on day 10 (2-way ANOVA, P<0.05). C) Probe trial on day 10. *Atxn2* ko mice spent an average of 33.5±3.0% (mean±SEM, n = 16) in quadrant 1 (Q1) compared to 23.7±2.9, 22.4±3.2, and 20.4±3.3% in Q2, Q3, and Q4, respectively. Wt mice (n = 15) spent 31.0±3.5 seconds in Q1 compared to 24.9±2.7, 24.1±2.9, and 20.0±2.7 in the other 3 quadrants. D) Probe trial on day 14. Both wild type and *Atxn2* ko mice spent significantly more time in quadrant 1 than other quadrants. *Atxn*2 ko mice spent an average of 39.5±2.9% in Q1 compared to 18.9±2.2, 21.5±2.9, and 20.3±2.7% in Q2, Q3, and Q4, respectively. Wild type mice spent an average 33.3±2.9% in Q1, and 26.3±3.4, 20.4±2.2, and 20.0±2.4 in Q2, Q3, and Q4, respectively.

On the first day, the platform was visible and was easily located by all mice. From days 2 to 13, the platform was submerged. Performance was evaluated by measuring the latency of mice to find the hidden platform in quadrant 1. With training (Fig. 2A), the latency of finding the hidden platform decreased significantly (2-way repeated measures ANOVA, P<0.0001) for both wild type and ko animals. There was no difference between genotypes over the 13 days of training (2-way repeated measures ANOVA, P = 0.9).

Probe trials, where the hidden platform in quadrant 1 was removed, were performed on days 10 and 14 after the last training on that day. The average swim speeds (Fig. 2B) for *Atxn2* ko mice and wild type littermates were similar on day 14. On day 10 (Fig. 1C), only *Atxn2* ko mice showed some evidence of learning (2-way ANOVA, P<0.009, Bonferroni posttests, P<0.05 for *Atxn2* ko, and P>0.05 for wt). This was further confirmed by one sample t-test assuming a hypothetical mean of 25% time spent in each of the 4 quadrants showing that only *Atxn2* ko mice spent more time in quadrant 1 on day 10 (one sample t-test, P = 0.025 for *Atxn2* ko, and P = 0.21 for wt mice). On day 14 (Fig. 2D), both wild type and *Atxn2* ko mice showed improved performance (2-way ANOVA, P<0.0001, Bonferroni posttests, p<0.001 for *Atxn2* ko and p<0.05 for wt). Both wild type and *Atxn2* ko mice spent a significantly higher percentage of time in quadrant 1 compared to other quadrants (one sample t-test, p = 0.03 for wt; p = 0.0002 for *Atxn2* ko).

### Fear conditioning is impaired in homozygous *Atxn2* ko mice

**Figure 3:**
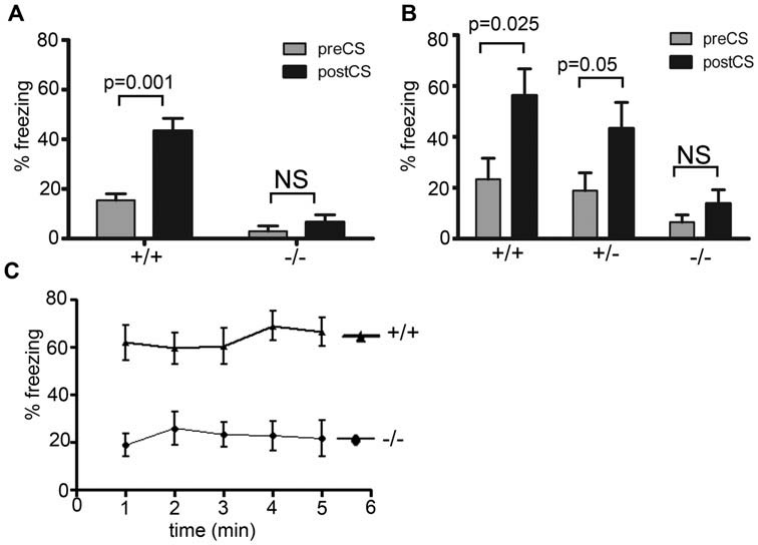
Reduced fear learning in two independent cohorts of *Atxn2* ko mice at 6 months of age. A) Cued conditioning of cohort A. The average pre-/post tone freezing was 15.4±2.6% (mean±SEM)/43.4±5.0% for wt (n = 22) compared to 3.0±2.1/6.6±2.9 for *Atxn2* ko mice (n = 16); the significance levels for pairwise comparisons for one-tailed Wilcoxon Signed Rank (WSR) tests adjusted for multiple comparisons are shown, NS = not significant. B) Cued fear conditioning of cohort B. The average pre-/post-tone freezing (mean±SEM) was 23.3±8.3/56.3±10.5 for wt (n = 7), 18.9±7.0/43.3±10.2 for heterozygote (n = 9), and 6.5±2.9/13.8±5.4 for *Atxn2* ko mice (n = 9); one-tailed WSR test significance levels adjusted for multiple comparisons are shown. C) Contextual test of mice in cohort A. *Atxn2* ko mice exhibited significant freezing deficits in contextual testing compared to wild type mice (2-way ANOVA with repeated measure, P<0.0001, Bonferroni post tests P<0.01 at 2 min and P<0.001 at all other time points). The average contextual freezing (mean±SEM) was 63.5±1.8% for wt (n = 22) compared to 22.4±1.1% for Atxn2 ko (n = 16) mice.

Auditory fear conditioning evaluates the ability of mice to remember an association between a tone and an electrical shock (Fig. 3A–B). Mice react to the electrical shock by freezing. For cued conditioning, mice are placed in a different environment 24 hours later and exposed to the tone alone. A learned association between the tone and the electrical shock manifests as increased freezing upon hearing the tone alone. We tested two cohorts of mice (cohorts A and B) and in both, baseline freezing prior to application of the electric shock was different in wt and ko animals. Each group of animals showed a wide range of behavioral responses which ranged from no freezing to >30% freezing (Supplemental Table S1). Differences in baseline freezing were likely related to the overall motor hyperactivity seen in ko animals (Fig. 1). We therefore evaluated the change in freezing pre- and post-conditioning in each animal using a paired test. As values were not normally distributed with different standard deviations in groups, we employed the paired Wilcoxon Signed Rank test (WSR) for statistical analysis.

Wt littermates in both cohorts showed cued fear conditioning and exhibited significantly increased freezing behavior (one-tailed WSR test p = 0.001 for cohort A, p = 0.025 for cohort B). Heterozygous mice (*Atxn2*+/−) in cohort B also exhibited a significant increase in freezing behavior, although to a lesser extent (one-tailed WSR test p = 0.05). In contrast, homozygous *Atxn2* ko mice showed reduced fear learning and the observed small increase in freezing was not significant in either cohort.

Contextual fear conditioning is evaluated by returning the mice to the environment in which they were exposed to the electrical shock. Mice that have learned the association between the training environment and the electrical shock will exhibit increased freezing. To test for contextual learning, mice from cohort A were placed in the same cage that had been used for conditioning for 5 minutes. Wild type mice froze immediately when they were placed in the conditioning box. In contrast, *Atxn2* ko mice showed only minimal freezing (Fig. 3C).

**Figure 4:**
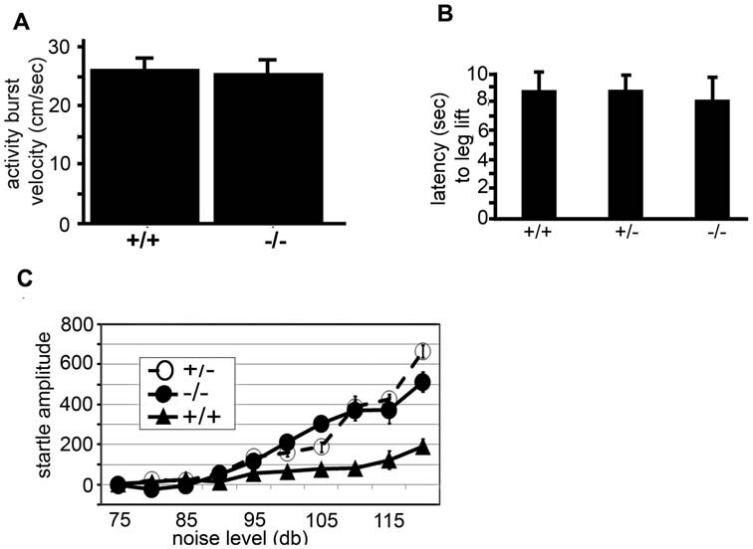
Unconditional, startle and hot plate responses are similar in knockout and wild type mice. A) No difference in activity burst velocity during the 2 sec following electric shock of wild type (n = 22) and *Atxn2* ko (n = 16) mice in cohort A. Activity burst velocity was determined as described in Anagnostaras et al, 2000 (19). B) Hot plate latency of wild type (n = 7), *Atxn2* heterozygous (n = 9) and *Atxn2* ko mice (n = 9) in cohort B. Reaction to pain on the hot-plate was similar for the three genotypes. C) Startle responses to increasing sound levels of wild type (n = 9), heterozygous (n = 9), and *Atxn2* ko (n = 7) mice. Startle was increased in heterozygous and *Atxn2* ko mice starting at sound levels >95 db (one-way ANOVA, p = 0.01).

Freezing deficits may result from lack of associative learning, but also from deficits in primary sensory pathways. To determine the response to the electric shock (unconditional response) in the two genotypes, we digitally measured the response of mice following an electrical shock using NIH image (Fig. 4A). Both wild type and *Atxn2* ko mice had similar activity burst velocities (26.7±1.8 cm/sec for wt vs. 25.4±1.5 cm/sec for *Atxn2* ko), suggesting that *Atxn2* ko mice perceived the shock as well as their wild type littermates.

For further confirmation of normal nociceptive responses in *Atxn2* deficient mice, we determined responses to pain stimuli in the hot plate paradigm. Wild-type and *Atxn2* ko mice responded similarly to heat-mediated painful stimuli. The latencies for wild-type (n = 7), heterozygous (n = 9) and homozygous mice (n = 9) were 8.8±1.2 s, 8.8±0.9 s and 8.1±1.5 s, respectively (Fig. 4B).

To investigate whether *Atxn2* ko mice had normal hearing, we determined startle responses to increasing sound levels. The startle test did not reveal deficits in *Atxn2* ko mice (Fig. 4C). For tones >95 dB startle was more prominent in homozygous and heterozygous *Atxn2* knockout mice than in wild-type littermates. Thus, differences in fear behavior between mice of different genotypes could not be attributed to differences in primary auditory or pain perception.

### Innate fear is decreased in *Atxn2* ko mice

**Figure 5:**
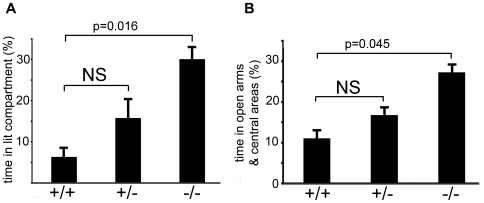
*Atxn2* ko mice exhibit significantly reduced innate fear compared to wild type littermates. A) Light-dark box test of wild type (n = 7), heterozygous (n = 9), and *Atxn2* ko (n = 9) mice. Homozygous *Atxn2* ko mice spent more time in the light compartment than their wild type littermates (30.0±4.3 vs. 6.3±4.4; Mann-Whitney (MW) test, two-tailed, p = 0.016, corrected for multiple comparisons). The performance of heterozygous mice was intermediate (15.7±12.4, MW-test vs. wt, p = 0.9). B) Elevated plus maze test. Homozygous mice spent significantly more time (27.3±5.0%) in the open arms and central zone compared to wild type (11.1±2.4%) littermates (MW, two-tailed, P = 0.045, corrected for multiple comparisons). Behavior of heterozygous mice was intermediate (16.8±5.3%, MW-test vs. wt, p = 0.88). Data are presented as mean±SEM. NS = not significant.

Homozygous *Atxn2* ko mice exhibited a lack of acquisition of fear conditioning (Fig. 3A–C). To examine whether this was paralleled by a decrease of innate fear or anxiety, mice were evaluated in the light-dark box assay and the elevated plus maze test. In the light-dark box assay (Fig. 5A), homozygous *Atxn2* ko mice spent significantly more time in the light compartment than their wild-type littermates (Mann-Whitney (MW), two-tailed, p = 0.016, corrected for multiple comparisons).

In another test of anxiety, the elevated plus maze, homozygous ko mice spent more than twice the time in the open arms and central zone compared with their wild type littermates (MW, two-tailed, p = 0.045). In both tests, heterozygous mice showed an intermediate behavior (Fig. 5B).

### Conditioned Taste Aversion (CTA)

**Figure 6:**
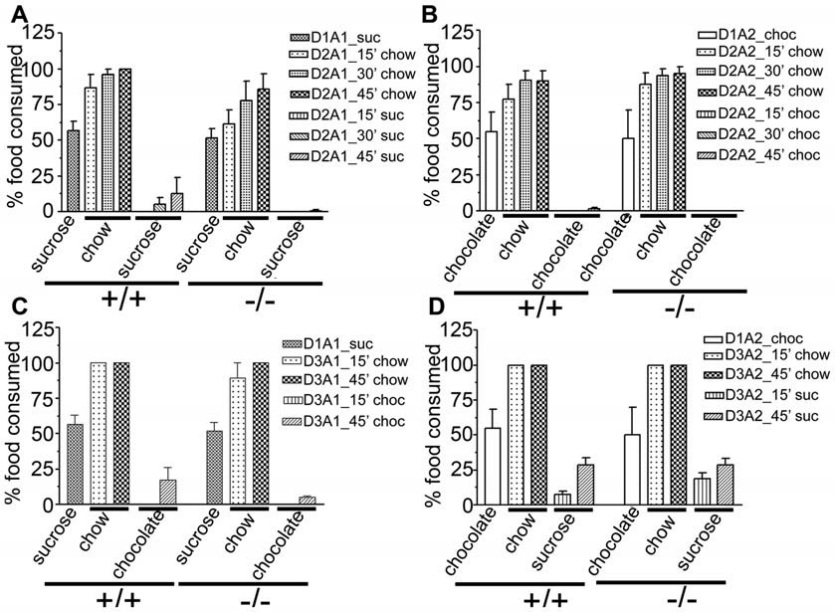
Normal behavior of *Atxn2* ko mice in the conditional taste aversion task. Panels A and B show results from the Conditional Taste Aversion test on days 1 and 2; panels C and D show the results from neophobia testing on day 3. On conditioning day 1, mice in group A1 (wt, n = 6; ko, n = 7) were exposed to regular chow and sucrose, whereas mice in group A2 (wt, n = 8; ko, n = 5) were exposed to chow and chocolate. Wild type mice consumed 56.7%±6.7 of sucrose pellets and 55%±13.3 (n = 8) of chocolate pellets. *Atxn2* ko mice consumed 51.3%±6.3 and 50.0%±20.0 of sucrose or chocolate pellets, respectively. A) Group A1 mice exhibited strong taste aversion to sucrose pellets on the testing day. At 45 min, wt and *Atxn2* ko mice consumed 100±0.0% (mean±SEM) and 85.7±10.7% of rodent chow pellets, respectively, while only 12.5±11.5% and 0.7±0.7% of sucrose pellets were consumed by wt and *Atxn2* ko mice, respectively. B) Group A2 mice showed strong taste aversion to chocolate pellets. At 45 min, wt and tax ko mice consumed 90.0±6.6% and 95.0±5.0% of rodent chow pellets, respectively. C, D) Neophobia test of wild type and *Atxn2* ko mice. Mice from group A1 (C) were fed chocolate-coated pellets, and conversely animals in group A2 (D) were fed sucrose pellets. Animals in both groups showed evidence of neophobia, irrespective of genotype. Percentage of food pellets (chow, sucrose, and chocolate) consumed at 15 and 45 minutes are shown. Differences between wild type and *Atxn2* ko mice were not significant (2-way ANOVA, P>0.05).

A number of different approaches have indicated that establishment of CTA depends on a network of brain regions that include the amygdala, medial thalamus, and insular cortex, whereas the hippocampus appears to play only a minor role [21]-[23]. Both wild type and *Atxn2* ko mice consumed similar amounts of sucrose or chocolate coated pellets prior to LiCl injection (Fig. 6). On day 2, the conditioned taste aversion test was conducted (Fig. 6A–B). Mice were offered the choice of 20 chow pellets and either 20 sucrose pellets (group A1, Fig. 6A) or 20 chocolate coated-pellets (group A2, Fig. 6B). Both wild type and *Atxn2* ko mice immediately consumed a large number of chow pellets within the first 15 minutes, while only very few chocolate or sucrose pellets were consumed even at the end of 45 minutes. These differences were highly significant for both wild type and *Atxn2* ko mice (ANOVA with repeated measure, P<0.0001). Similar results were obtained for group A2 (2-way ANOVA with repeated measure, P<0.0001, Bonferroni posttests, P<0.001 for wt and P<0.05 for *Atxn2* ko mice). These observations suggested that both wt and *Atxn2* ko mice were able to learn an aversive taste equally well.

On the third day, an aversion to novel but unassociated food types (neophobia) was tested (Fig. 6C–D). Group A1 mice, originally fed with sucrose pellets, were now offered the choice of 20 pellets of chow or 20 pellets of chocolate (Fig. 6C). Group A2 mice, originally fed with chocolate pellets, were offered a choice of chow or sucrose pellets (Fig. 6D). Both wt and *Atxn2* ko mice exhibited a tendency to neophobia to chocolate when the CS had been sucrose ((2-way ANOVA, P<0.0001, Bonferroni post tests, P<0.001 for both wt and *Atxn2* ko). Neophobia to sucrose was present as well (2-way ANOVA, P<0.0001, Bonferroni post tests, P<0.001). There were no differences between genotypes (P>0.05). These observations suggested that both wt and *Atxn2* ko mice showed a generalization of taste aversion to novel tastes.

### Long-term potentiation is impaired in the amygdala but not in the hippocampus of *Atxn2* ko mice

**Figure 7:**
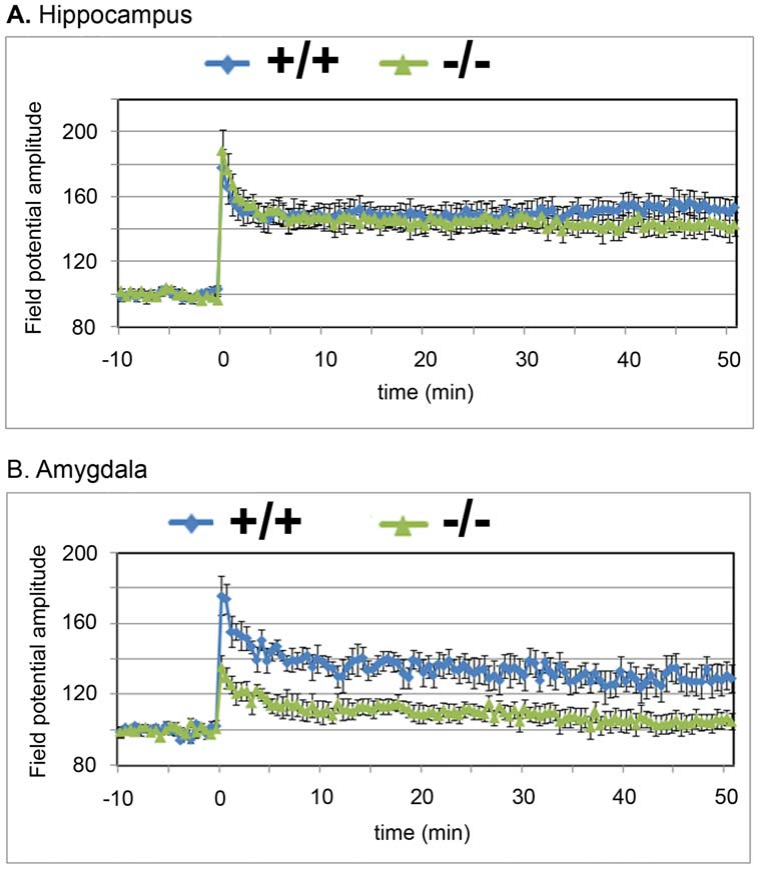
Long term potentiation (LTP) in the hippocampus and basolateral amygdala. A) LTP from 7 repeated experiments from hippocampal slices of 3 wild type (blue tracer) and *Atxn2* ko (green tracer) mice. LTP is normal in the hippocampus of *Atxn2* ko mice (one-away ANOVA, p = 0.35). B) LTP from 8 repeated experiments from amygdala slices of 4 wild type (blue) and 4 (green) *Atxn2* ko mice. *Atxn2* ko mice exhibited significant reduction of LTP amplitude compared to their wild type littermates (one-way ANOVA, P<0.05). Each time point represents the measured change in amplitude of the field potential (mean±SEM).

Previous studies have shown that decreases in LTP are associated with deficits in fear conditioning and hippocampal spatial learning. To test this in *Atxn2* ko mice, the ability to induce LTP was measured in slices from the hippocampus and the amygdala. Hippocampal LTP was not significantly different between wt and *Atxn2* ko mice (Fig. 7A). High-frequency stimulation of the Schaffer collaterals increased the CA1 field potential amplitude by 53±7% in wild-type mice (n = 7 from 3 mice) and by 43±7% in homozygous knockout mice (n = 7 from 3 mice).

In the amygdala, field potentials were recorded in the basolateral nucleus in response to high-frequency stimulation in the dorsolateral amygdala (adjacent to the external capsule). In wild type mice, the amplitude of the field potential increased by 29±8% (n = 8 from 4 mice). In contrast, homozygous knockout littermates exhibited only a 5±5% increase (n = 8 from 4 mice) in the amplitude of the field potential (Fig. 7B). The LTP differences between wild type and homozygous *Atxn2* ko mice were statistically significant (one-way ANOVA, p<0.05). Although the observations of differential hippocampal and amydalar LTP are interesting, these data should be interpreted carefully as the numbers of animals used in these studies are small. Regardless, these electrophysiological data are consistent with current interpretation of the behavioral findings.

## Discussion

Genetic variation in the *ATXN2* gene has been linked to human disease and to adaptive selection. Mutations in the form of expansions of the CAG DNA repeat to ≥32 repeats cause spinocerebellar ataxia type 2, a human neurodegenerative disease. On the other hand, a specific CAG/CAA repeat allele, (CAG)_8_CAA(CAG)_4_CAA(CAG)_8_, is highly selected in Northern Europeans suggesting adaptive advantages conferred by this particular repeat structure [24]. Delineating behavioral consequences of *Atxn2* deficiency in the mouse is thus important for understanding pathology in SCA2 patients and selective forces in Northern Europeans.

We report significant and specific behavioral abnormalities in *Atxn2*-deficient mice accompanied by impaired LTP in the amygdala and normal LTP in the hippocampus. Behavioral changes encompassed motor hyperactivity, reduced signs of innate fear/anxiety and associative fear learning, but did not extend to other associative learning paradigms such as learning of spatial cues or aversive tastes. Thus, *Atxn2* knockout mice may represent an additional rare example of dissociated hippocampal and amygdalar synaptic plasticity [25]–[27].

### Fear

The salient behavioral abnormalities in *Atxn2* ko mice at 6 months of age were reduced innate and learned fear and motor hyperactivity. Of these, only motor hyperactivity had been previously described in an independently generated ko mouse line [15]. During open cage testing, we observed that in one of our cohorts, *Atxn2* ko animals spent more time in the center of the open cage, a potential indication of reduced anxiety. We therefore explored behaviors related to innate fear and anxiety in the elevated plus maze and the light/dark box paradigms. In both tests, *Atxn2* ko mice showed signs of reduced fear and anxiety with heterozygotes exhibiting an intermediate phenotype.

Abnormalities extended also to associative fear learning. Fear conditioning is a primal form of learning that is mediated by the amygdala [28], [29]. During fear conditioning, an innocuous sensory stimulus, most frequently a tone, acquires the ability to elicit defensive behavioral responses such as freezing after being paired with a threatening stimulus, usually an electric shock. In two independent groups of mice tested by two investigators (DH and MM), *Atxn2* ko mice showed greatly reduced freezing. We excluded primary auditory and nociceptive abnormalities by showing normal auditory startle, hot plate performance, and similar jump velocity following the electric shock (Fig. 4C–D).

Anatomical, physiological and behavioral studies have suggested that the learned association between tone and foot shock occurs in the basolateral complex, the input region of the amygdala. The synaptic changes underlying increased neuronal responsiveness to auditory stimuli are mechanistically similar to LTP as pharmacological agents that impair fear conditioning inhibit LTP in the amygdala as well [30]. Transgenic mice with impaired LTP in the amygdala do not exhibit fear conditioning [31], [32]. Furthermore, fear conditioning increases LTP activity in rat amygdala [33], [34]. LTP impairment in the amygdala of *Atxn2* ko mice is consistent with defects in the neuronal circuits underlying fear-related behaviors associated with amygdalar processing. Interestingly, hyperphagia, which has been observed in *Atxn2* ko mice, can also be seen with lesions in the amygdala [35].

Changes in amygdalar plasticity were not generalized to other structures. LTP in the hippocampus was normal (Fig. 7A) and two other tests of learning were normal as well. Both *Atxn2* ko and wild type mice spent a significantly greater amount of time in the probe quadrant (Q1) than other quadrants in the hippocampus dependent Morris water maze test (Fig. 4). In a second test of associative learning, the conditional taste aversion paradigm, *Atxn2* ko mice performed as well as their wild type littermates (Fig. 6). This was somewhat surprising as fear conditioning and CTA both involve plasticity in the amygdala. However, cued fear conditioning is largely mediated through the basolateral complex of the amygdala, whereas taste aversion involves the central nucleus [36].

### Differential Plasticity

Differential hippocampal and amygdalar plasticity has been observed in a relatively small number of mouse models. Shumyatski et al identified two amygdala-enriched proteins, stathmin and gastrin-related peptide (GRP) [26], [27]. Knockout of stathmin resulted in deficient amygdalar LTP and reduced cued and contextual fear conditioning as well as increased exploration of the open arm in the elevated plus maze. Knockout of the GRP receptor increased cued and contextual fear memories but left spatial learning unaffected. This was accompanied by enhanced cortico-amygdalar LTP. Unfortunately, CTA was not examined in these mouse strains.

In contrast to stathmin and GRP receptor, ataxin-2 is not known to show differential regional expression except for enhanced expression in cerebellar Purkinje cells and some hippocampal neurons [5]. It is possible; however, that a subset of ataxin-2 interacting proteins shows regionally restricted expression. Although ataxin-1 and ataxin-2 interact physically and genetically [37.38], the knockout phenotypes of the respective genes show little overlap [16] with the caveat that innate and learned fear behaviors were not reported for *Atxn1* ko mice.

### Relationship to SCA2 phenotype

Our study may guide future clinical investigations of the SCA2 phenotype in humans. If mutant *Atxn2* does not only lead to gain of a toxic polyQ function, but also or even exclusively to a gain of normal function, then, despite the caveats of mouse-man comparisons, the study of symptomatic and presymptomatic SCA2 mutation carriers may potentially be informed by our studies. Thus, it would be interesting to examine activity and anxiety levels in presymptomatic and symptomatic SCA2 mutation carriers as well as specific spatial learning tasks. It is already known that executive function is abnormal and altered early in the course of SCA2 [1], [2], but of course precisely these cognitive functions remain difficult to study in rodents.

### Conclusions

In conclusion, our behavioral studies lay the groundwork for studying Atxn2 function in a number of neurological diseases. Atxn2 has not only been implicated in human neurodegenerative disease, but it remains a candidate for autistic spectrum disorders and bipolar disorder due to its interaction with A2BP1/Fox-1 [10], [39]. Hemizygous disruption of A2BP1/Fox-1 by chromosomal translocation events causes behavioral phenotypes in humans [39], [40]. Fear-related behavioral changes in heterozygous and homozygous *Atxn2* knockout mice are consistent with these observations in humans. Central Europeans exhibit selection of specific *ATXN2* CAG-repeat alleles that do not result in a different protein sequence, but may affect transcript or protein abundance (40). In this context, it is important to note that we observed gene-dosage dependent behavioral effects as heterozygote mice showed fear-related behaviors that were intermediate between wt and knockout animals. This dosage sensitivity suggests the *ATXN2* gene as a good candidate for detailed analysis of genetic variation, both common and rare, in a number of human phenotypes as diverse as obesity, motor hyperactivity, anxiety, and depression.

## Supporting Information

Figure S1 Male and Female Atxn2 ko mice exhibited similar behavior deficits in Open-field and Fear Conditioning Tests. Behavior analyses of cohort A male and female Atxn2 ko mice. Panels A and B show the Open Field tests of male and female Atxn2 ko mice. A total of 7 wild type male, 16 wild type female, 8 male Atxn2 ko and 9 female Atxn2 ko mice were used for Open Field experiments. Panel A and B show the locomotive activities in the Open Field experiments of male and female wild type and Atxn2 K0 mice, respectively. Both male and female Atxn2 ko mice exhibit increased locomotive activities compared to their respective gender wild type mice (one-way ANOVA, P<0.0001, Tukey Multiple Comparison Test, P<0.05). C).Pre- and post conditional stimulus (preCS and postCS) of cohort A male and female mice. A total of 13 wt female, 9 wt male, 9 female Atxn2 ko, and 7 male Atxn2 ko were used for Cued and Contextual Fear Conditioning. Since the data were randomly scattered, we used one-tailed, Mann-Whitney test to compare differences between male and female mice. Both male and female Atxn2 ko mice did not show any significant freezing compared to wild type mice. Both female and male wild type mice showed significant increasing in freezing after post conditional stimulus (preCS vs. postCS female wt, P<0.0001; male wt, P<0.01). There was no significant difference between preCS and postCS of both cohort A female and male Atxn2 ko mice as Atxn2 ko mice exhibited very little freezing activity during postCS. Although male wt mice exhibited significantly lower freezing activity than female wt mice during post conditional stimulus (Mann-Whitney test, P<0.01), both male and female Atxn2 ko mice exhibited significantly lower freezing activity compared to the female wild type mice (Mann-Whitney test, P<0.004 during postCS of female wt vs. female Atxn2 ko; P<0.02 during postCS of male wt vs. female Atxn2 ko). Since the SD of the male Atxn2 ko mice was zero, the statistic could not be compared between male wt and male Atxn ko mice).
(1.23 MB TIF)

Figure S2 Open-field test of cohort B Atxn2 ko mice. A) Average speed of wild type (n = 7), heterozygous (n = 9), and homozygous (n = 9) Atxn2 mice. Average speed (cm/sec, Mean±SEM) of wild type, heterozygous, and homozygous mice were 15.65±1.15, 15.11±1.07, and 21.03±0.84, respectively. Unlike cohort A mice, Atxn2 ko mice in cohort B traveled at a higher speed than either the wild type or Atxn2 heterozygous mice (repeated measures, one-way ANOVA, P<0.0001). B) Average ambulatory distances traveled by group B mice during 20 minutes in the open cage. The average ambulatory distances (mean±SEM) traveled by wild type (+/+), heterozygous (+/−), and homozygous (−/−) mice were 59.88±6.75, 58.00±7.59, and 104.7±8.63, respectively. Similar to cohort A mice, cohort B Atxn2 ko mice traveled greater distances than either the wild type or Atxn2 heterozygous ko mice (Friedman test 1, one-way ANOVA, P<0.0001). C) Zonal distribution of group B mice. Unlike cohort A mice, cohort B Atxn2 ko mice preferred the central zone over the peripheral zone (568.22+50.84 sec vs. 381.00±57.95 (wt), repeated measures, 2-way ANOVA, P<0.03, Bonferroni posttest, P<0.05). Although wild type and heterozygous mice spent relatively more time in the center than in the peripheral zone (303.00±44.47 sec vs. 258.71±67.65 sec, P>0.05) and Atxn2 heterozygous (268.78±43.52 vs. 252.67±36.82 sec, P>0.05), times spent in the center and the peripheral zone were not significantly different.
(0.54 MB TIF)

Table S1 Raw data of Fear conditioning tests for cohort A and B. Data of fear conditioning tests for cohort A and B. Atxn2 ko mice. wt, het and hom signify wild-type (+/+), heterozygous (+/−), and homozygous (−/−) mice respectively. PreCS and postCS refer to preconditioning and post conditioning stimulus, respectively. Note that some of the wt mice do not show freezing at baseline, but all of these do show substantially increased freezing after conditioning.
(0.03 MB XLS)
