# Supplementary figures and images for: Dissociated Fear and Spatial Learning in Mice with Deficiency of Ataxin-2
Source: PLoS One. 2009 Jul 20;4(7):e6235. doi: 10.1371/journal.pone.0006235 (PMC2707006; doi:10.1371/journal.pone.0006235)

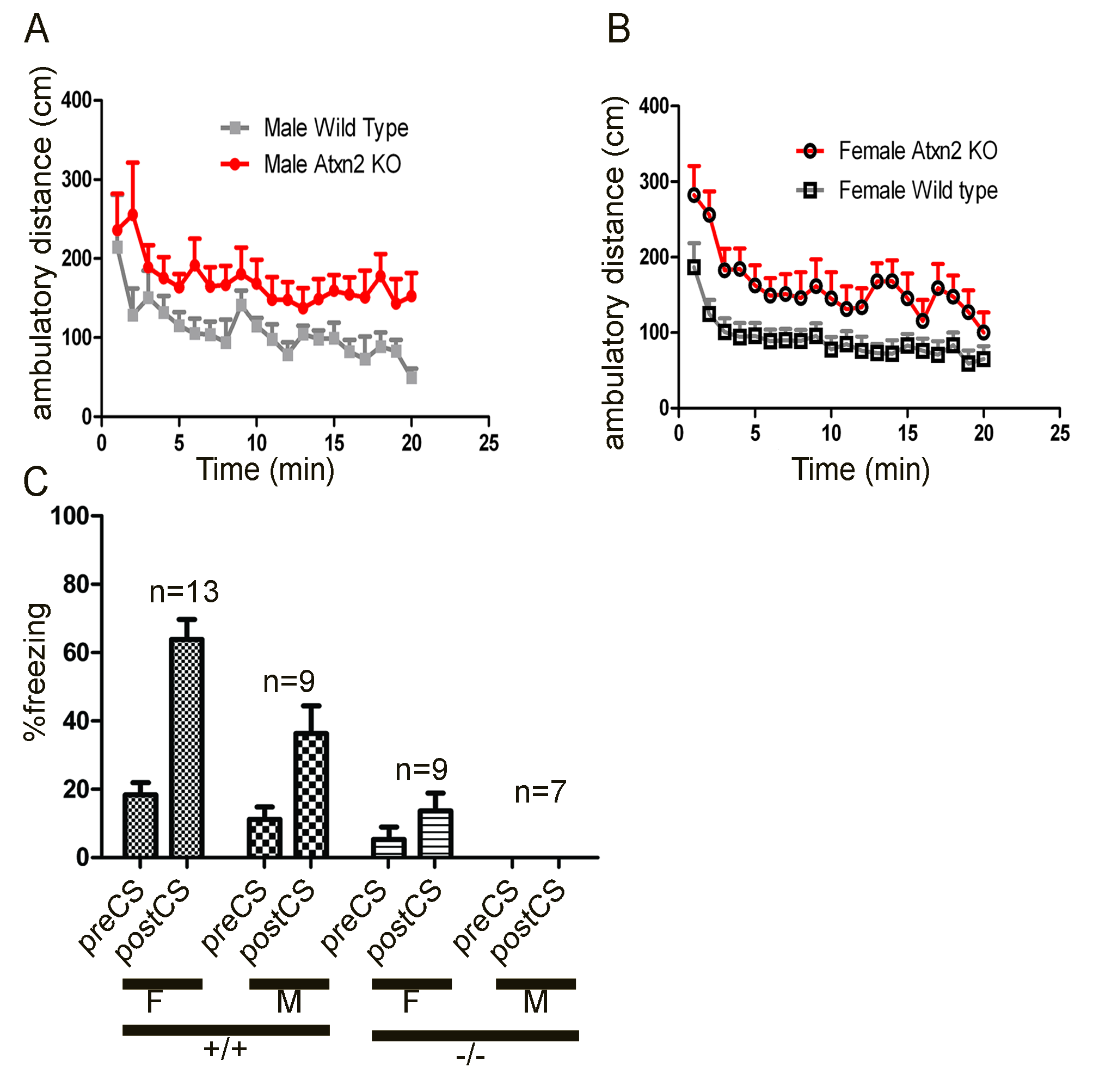

Supplement: Figure S1 — Male and Female Atxn2 ko mice exhibited similar behavior deficits in Open-field and Fear Conditioning Tests. Behavior analyses of cohort A male and female Atxn2 ko mice. Panels A and B show the Open Field tests of male and female Atxn2 ko mice. A total of 7 wild type male, 16 wild type female, 8 male Atxn2 ko and 9 female Atxn2 ko mice were used for Open Field experiments. Panel A and B show the locomotive activities in the Open Field experiments of male and female wild type and Atxn2 K0 mice, respectively. Both male and female Atxn2 ko mice exhibit increased locomotive activities compared to their respective gender wild type mice (one-way ANOVA, P<0.0001, Tukey Multiple Comparison Test, P<0.05). C).Pre- and post conditional stimulus (preCS and postCS) of cohort A male and female mice. A total of 13 wt female, 9 wt male, 9 female Atxn2 ko, and 7 male Atxn2 ko were used for Cued and Contextual Fear Conditioning. Since the data were randomly scattered, we used one-tailed, Mann-Whitney test to compare differences between male and female mice. Both male and female Atxn2 ko mice did not show any significant freezing compared to wild type mice. Both female and male wild type mice showed significant increasing in freezing after post conditional stimulus (preCS vs. postCS female wt, P<0.0001; male wt, P<0.01). There was no significant difference between preCS and postCS of both cohort A female and male Atxn2 ko mice as Atxn2 ko mice exhibited very little freezing activity during postCS. Although male wt mice exhibited significantly lower freezing activity than female wt mice during post conditional stimulus (Mann-Whitney test, P<0.01), both male and female Atxn2 ko mice exhibited significantly lower freezing activity compared to the female wild type mice (Mann-Whitney test, P<0.004 during postCS of female wt vs. female Atxn2 ko; P<0.02 during postCS of male wt vs. female Atxn2 ko). Since the SD of the male Atxn2 ko mice was zero, the statistic could not be compared between [file pone.0006235.s001.tif]

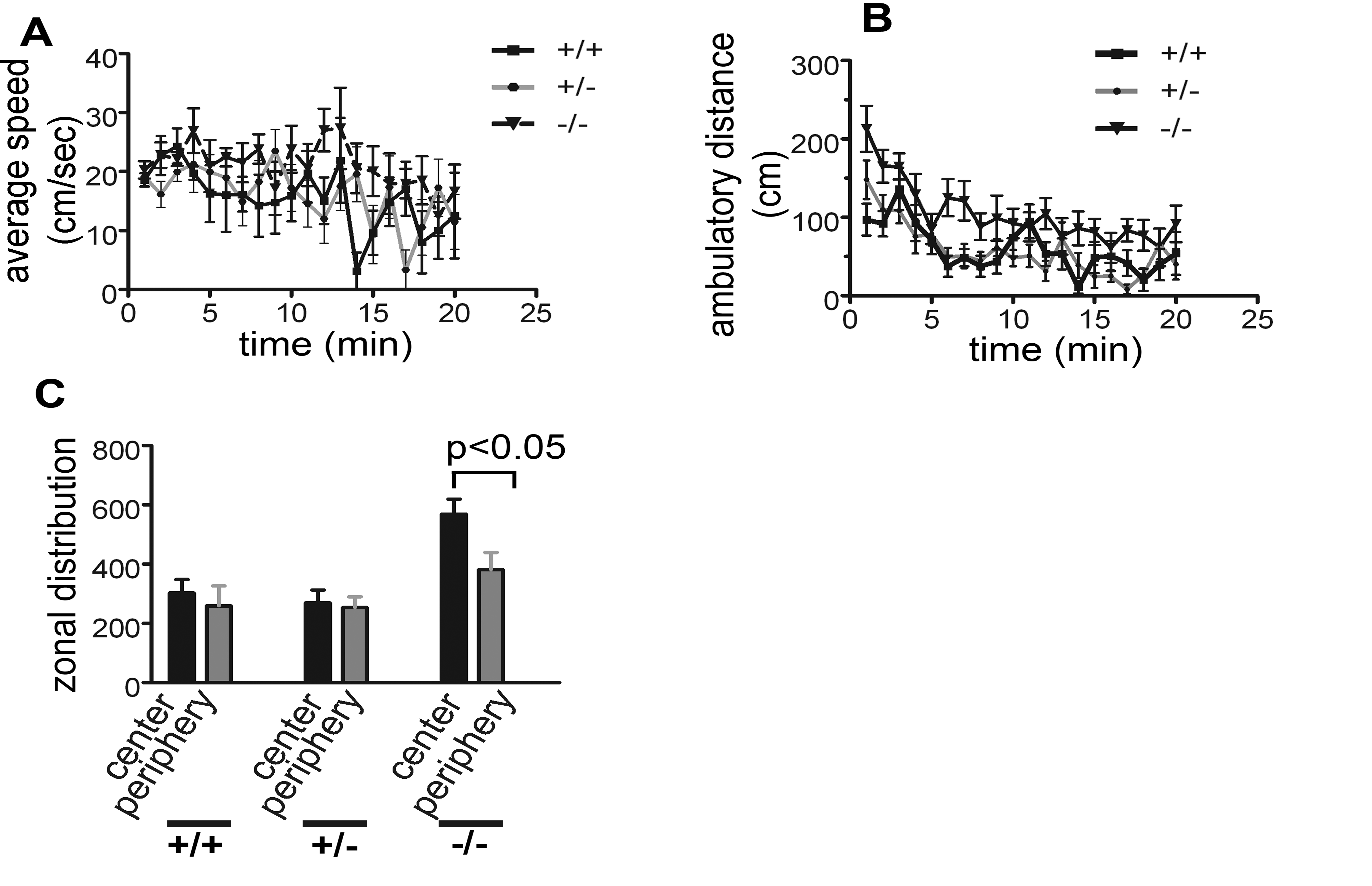

Supplement: Figure S2 — Open-field test of cohort B Atxn2 ko mice. A) Average speed of wild type (n = 7), heterozygous (n = 9), and homozygous (n = 9) Atxn2 mice. Average speed (cm/sec, Mean±SEM) of wild type, heterozygous, and homozygous mice were 15.65±1.15, 15.11±1.07, and 21.03±0.84, respectively. Unlike cohort A mice, Atxn2 ko mice in cohort B traveled at a higher speed than either the wild type or Atxn2 heterozygous mice (repeated measures, one-way ANOVA, P<0.0001). B) Average ambulatory distances traveled by group B mice during 20 minutes in the open cage. The average ambulatory distances (mean±SEM) traveled by wild type (+/+), heterozygous (+/−), and homozygous (−/−) mice were 59.88±6.75, 58.00±7.59, and 104.7±8.63, respectively. Similar to cohort A mice, cohort B Atxn2 ko mice traveled greater distances than either the wild type or Atxn2 heterozygous ko mice (Friedman test 1, one-way ANOVA, P<0.0001). C) Zonal distribution of group B mice. Unlike cohort A mice, cohort B Atxn2 ko mice preferred the central zone over the peripheral zone (568.22+50.84 sec vs. 381.00±57.95 (wt), repeated measures, 2-way ANOVA, P<0.03, Bonferroni posttest, P<0.05). Although wild type and heterozygous mice spent relatively more time in the center than in the peripheral zone (303.00±44.47 sec vs. 258.71±67.65 sec, P>0.05) and Atxn2 heterozygous (268.78±43.52 vs. 252.67±36.82 sec, P>0.05), times spent in the center and the peripheral zone were not significantly different. (0.54 MB TIF) [file pone.0006235.s002.tif]
